# The G Protein-Coupled Receptor Heterodimer Network (GPCR-HetNet) and Its Hub Components

**DOI:** 10.3390/ijms15058570

**Published:** 2014-05-14

**Authors:** Dasiel O. Borroto-Escuela, Ismel Brito, Wilber Romero-Fernandez, Michael Di Palma, Julia Oflijan, Kamila Skieterska, Jolien Duchou, Kathleen Van Craenenbroeck, Diana Suárez-Boomgaard, Alicia Rivera, Diego Guidolin, Luigi F. Agnati, Kjell Fuxe

**Affiliations:** 1 Department of Neuroscience, Karolinska Institutet, Retzius väg 8, 17177 Stockholm, Sweden; E-Mails: ismel@iiia.csic.es (I.B.); wromfdez@gmail.com (W.R.-F.); michael.dipalma@uniurb.it (M.D.P.); luigiagnati@tin.it (L.F.A.); 2 IIIA-CSIC, Artificial Intelligence Research Institute, Spanish National Research Council, 08193 Barcelona, Spain; 3 Department of Earth, Life and Environmental Sciences, Section of Physiology, Campus Scientifico Enrico Mattei, Urbino 61029, Italy; 4 Department of Physiology, Faculty of Medicine, University of Tartu, Tartu 50411, Estonia; E-Mail: juliaofli@gmail.com; 5 Laboratory of Eukaryotic Gene Expression and Signal Transduction (LEGEST), Ghent University, 9000 Ghent, Belgium; E-Mails: kamila.skieterska@ugent.be (K.S.); jolien.duchou@ugent.be (J.D.); kathleen.vancraenenbroeck@ugent.be (K.V.C.); 6 Department of Cell Biology, School of Science, University of Málaga, 29071 Málaga, Spain; E-Mails: boomgaard@uma.es (D.S.-B.); arivera@uma.es (A.R.); 7 Department of Molecular Medicine, University of Padova, Padova 35121, Italy; E-Mail: diego.guidolin@unipd.it

**Keywords:** G protein-coupled receptors, network, heterodimerization, heteromers, dimerization, oligomerization, hubs, receptor-receptor interactions, clusters, architecture

## Abstract

G protein-coupled receptors (GPCRs) oligomerization has emerged as a vital characteristic of receptor structure. Substantial experimental evidence supports the existence of GPCR-GPCR interactions in a coordinated and cooperative manner. However, despite the current development of experimental techniques for large-scale detection of GPCR heteromers, in order to understand their connectivity it is necessary to develop novel tools to study the global heteroreceptor networks. To provide insight into the overall topology of the GPCR heteromers and identify key players, a collective interaction network was constructed. Experimental interaction data for each of the individual human GPCR protomers was obtained manually from the STRING and SCOPUS databases. The interaction data were used to build and analyze the network using Cytoscape software. The network was treated as undirected throughout the study. It is comprised of 156 nodes, 260 edges and has a scale-free topology. Connectivity analysis reveals a significant dominance of intrafamily *versus* interfamily connections. Most of the receptors within the network are linked to each other by a small number of edges. DRD2, OPRM, ADRB2, AA2AR, AA1R, OPRK, OPRD and GHSR are identified as hubs. In a network representation 10 modules/clusters also appear as a highly interconnected group of nodes. Information on this GPCR network can improve our understanding of molecular integration. GPCR-HetNet has been implemented in Java and is freely available at http://www.iiia.csic.es/~ismel/GPCR-Nets/index.html.

## 1. Introduction

A large number of cellular processes are mediated through physical protein-protein interactions. Protein association is implicated in cellular signal transduction, regulation of gene expression, post-translational modification, and in protein function. A huge variety of protein associations are multimeric in their biological active state [1]. Hence, extensive research was carried out to identify and to understand the underlying principles of protein association based on the postgenomic emerging concept that the cell must be viewed as complex networks of interacting biomolecules instead of individual cellular components with their own, specific functions [2–5].

Despite the seemingly vast differences among these cellular networks, they all share common features in terms of network topology. The topological analysis of large networks of biomolecule interactions has contributed to the functional prediction of biological and pharmacological propensities of novel genes or proteins [6–8]. However, several functions of proteins have yet to be fully elucidated or even predicted.

Networks have proven to be a useful mathematical artifact for studying complex systems in multiple disciplines such as economics, sociology, and biology [2,6,8,9]. The term biological network refers to networks that describe relationships among a set of elements within biological systems. A network can be formally defined by a set of *N* elements and a set of *R* relations among those elements. A graph is a common way of visualizing networks. In a network graph, the nodes of the graph are the elements of the network and the edges correspond to the relations among the elements of the network. Knowing the model of a biological network is essential to further understand the complex system that is modeled. To this aim we need to examine some topological features like, for example, the node degree distribution and the clustering coefficient. The most elementary topological feature of a network is the node degree, which measures the number of connections or links the node maintains with other nodes. Conversely, the analysis of the clustering coefficient assesses the trend of the nodes of the network to form clusters.

One of the most unpredictable and confounding post-translational protein functions is the heterodimerization of G protein-coupled receptors (GPCRs). The description of a GPCR superfamily expanded considerably after the important discovery by Lefkowitz and colleagues in 1986 [10]. A wide range of GPCRs was proven to function not only as homomers but also as heteromers [11–18]. In the past, interactions between receptors were regarded only as a result of interactions due to changes in membrane polarization or to changes in phosphorylation/dephosphorylation of receptors. However, our observations in the early 1980s emphasized the existence of direct receptor–receptor interactions in the plasma membrane between different types of GPCRs [19–22]. As a logical consequence of the indications of direct physical interactions between neuropeptide and monoamine receptors, we introduced the term heterodimerization in 1993 to describe a specific direct interaction between different types of GPCRs [23]. The concept of a GPCR heterodimer was later confirmed in 1998–1999 by studies reporting that two non-functional GPCR monomers, GABA_B1_ and GABA_B2_, can assemble in a signaling heterodimer [24]. The GABA_B_ receptor belongs to the class C GPCR with heterodimerization taking place between the Venus flytrap modules and the *C*-terminal coiled-coil domains [25–27]. At the beginning of this century, a series of important contributions have confirmed the relevance of dimerization processes within the GPCR superfamily; special mention is the pioneering work of the Fuxe [28], Franco [29,30], Bouvier [31–33], Reynolds [34–36], Devi [37], Kenakin [38], George [39,40], Wess [41], Blumer [42], Bockaert [43] and Portoghese [44,45] groups, as some relevant examples.

Allosteric mechanisms make possible the integrative activity intermolecularly via receptor–receptor interactions in GPCR homomers, heteromers and receptor mosaics (higher order oligomers) [11,13–16,46–51]. Receptor–receptor interactions markedly increase the repertoire of GPCR recognition, signalling and trafficking in receptor heteromers. The GPCR assemblies mentioned are not isolated but usually also directly interact with other proteins with which they form the horizontal molecular networks in the plasma membrane.

Direct interactions involving GPCRs were demonstrated through diverse methods that assess receptor–receptor interactions [12,17,52–59]. In this study, we manually collect static/non-dynamical human GPCR data derived from these interaction studies in annotated databases and literature. We further integrate the relationship information in a large-scale graph, called the GPCR heterodimer network [60], where the vertices are the receptor protomers and the edges are their relationships. The results from the GPCR-HetNet indicate a scale-free model in which a few of the protomers dominate the connectivity and hold the network together. Three different hub criteria show that the dopamine D2 receptor (DRD2), the beta-2 adrenergic receptor (ADRB2), the growth hormone secretagogue receptor type 1 (GHSR), the mu-type opioid receptor (OPRM), the delta-type opioid receptor (OPRD), the kappa-type opioid receptor (OPRK), the adenosine A2A receptor (AA2AR) and the adenosine A1 receptor (AA1R) are the hubs in the network. Other highly connected protomers are also identified and described in this study, as well as the emergence of potential allosteric mechanism avenues and higher order heteroreceptor complexes. In this study we present for the first time the overall architecture of the GPCR heteromers. The GPCR-HetNet provides insight into receptor–receptor interaction connectivity, topology, and organization that could be used to generate plausible hypotheses and help researchers to better understand GPCR heteromer systems and design experiments.

## 2. Results and Discussion

### 2.1. The GPCR-HetNet Dataset

Scopus (http://www.scopus.com) and the Search Tool for the Retrieval of Interacting Genes (STRING: http://string-db.org/) databases were searched for experimentally validated interactions of GPCR heteromers, supported by at least one detection method (among others co-immunoprecipitation [17], bioluminescent and fluorescent energy transfer methods (Bioluminescence Resonance Energy Transfer (BRET), Fluorescence Resonance Energy Transfer (FRET), Sequential Resonance Energy Transfer (SRET), Time-Resolved Fluorescence Resonance Energy Transfer (TR-FRET), and the Bimolecular Fluorescence Complementation (BiFC) approach) [14,53], fluorescence cross-correlation spectroscopy (FCCS) [57,58] and *in situ* Proximity Ligation Assays (PLA) [12]). Each interaction was inspected twice to confirm the literature information. Experimentally verified physical receptor–receptor interactions were reported for 156 GPCR protomers (Table 1), which collectively account for approximately 20% of the total number of putative human GPCR protomers (a total number of 797 human GPCRs exists in the UniProt database, as recently annotated and described by Jassal *et al.*, 2010 [61]). This percentage can be considered as a substantial sample of the GPCR population.

According to the UniProt classification, we were able to retrieve 128 rhodopsin-like protomers (class A, also known as Family 1) representing 18%–25% of the total number of putative protomers in this superfamily (726 including orphan receptors and 519 true non-orphan receptors, respectively). Interaction data for the rhodopsin-like superfamily members, which included non-orphan and orphan receptors, were the most incomplete although they represent approximately 82% of the total number of identified protomers. In comparison, experimentally verified interactions were reported for 15 out of 46 members of the Secretin-like superfamily members (class B, also known as Family 2) representing 33% of the total number of putative protomers of this superfamily; and 13 out of 22 metabotropic Glutamate receptor-like superfamily members (class C, also known as Family 3) were involved in at least one interaction, representing 60% of the total number of putative protomers of this superfamily (Table 1).

The network was built taking into account only GPCR heteromers. We excluded homomer information although it can be important to mention that more than 87% of the total identified protomers exist as homomers as well. It must be underlined that it was recently demonstrated using fluorescence correlation spectroscopy with photon counting histogram analysis (a sensitive method for monitoring diffusion and oligomer size of plasma membrane proteins) that biogenic amine receptors freely diffusing within the plasma membrane are predominantly homodimers and not monomers [58]. The balance between homo- *versus* heteromer GPCR populations is an important factor to take into account based on the fact that it could be the molecular determinant behind some pathological diseases where the GPCR dimerization phenomenon plays a role.

The analysis of receptor–receptor interaction (intrafamily and interfamily) connectivity reveals a significant dominance of intrafamily *versus* interfamily connections (Table 1). One mechanism that might explain such a marked difference could be a favourable co-evolution of the protomer interface interaction inside each subfamily. GPCRs have high sequence homologies/similarities inside each superfamily but reduced shared sequence homologies between them [62]. For example, the Secretin-like superfamily, one of the largest and best-studied hormone and neuropeptide receptor families, is suggested to have emerged from a single ancestral gene via duplication events. As a result, it shows a high sequence homology between its members [63]. It has been demonstrated that homologous proteins belonging to the same family can share similar interfaces where their exposed residues can be either intermixed or run in parallel to one another [64]. Also, their conserved domains or motifs can take part in domain swapping phenomena as shown for some GPCR heteromers [35,36,65]. A second reason could be the diversity/specificity in the cell and tissue expression pattern for some receptor clans. Some receptor classes are more widely expressed in some tissues or organs than others, and as a result allow their members to have a higher probability of encounters and interactions. A further reason that cannot be excluded is the lack of experimental data or analysis of GPCR interfamily heterodimerization. In the last decade few research groups have focused on the study of GPCR heterodimerization specificities, which may unravel a more widespread existence of cross-family heterodimerization; alternatively, it is possible that it will verify a substantial degree of intrafamily GPCR-GPCR specificities.

### 2.2. Analysis of the GPCR-HetNet Architecture: Network Measures and Models

The behaviour of the most complex biological systems emerges from the orchestrated activity of many components that interact between each other through pairwise or multiple connections. These components can be reduced to a series of nodes and edges that form a network or, in more formal mathematical language, a graph. Establishing the identity of the GPCR receptor–receptor interaction networks is not trivial but physical interactions and connection between GPCR protomers can easily be conceptualized using the node-edge nomenclature, where the edges do not have an assigned direction. In the GPCR-HetNet, where the edge represents a mutual protomer binding relationship, it follows that if protomer A binds to protomer B, then protomer B also binds to protomer A. But how can this network be characterized? Networks are typically evaluated at two levels: the topology, which describes the architecture of the graph, as well as the interactions within. Network topology plays a vital role in understanding and evaluating the network architecture and performance. Several of the most important topological metrics include node degree distribution, clustering coefficient, and path length, which allow us to characterize different complex networks. Detailed descriptions of these metrics are listed in the Experimental section.

**Figure 1.**
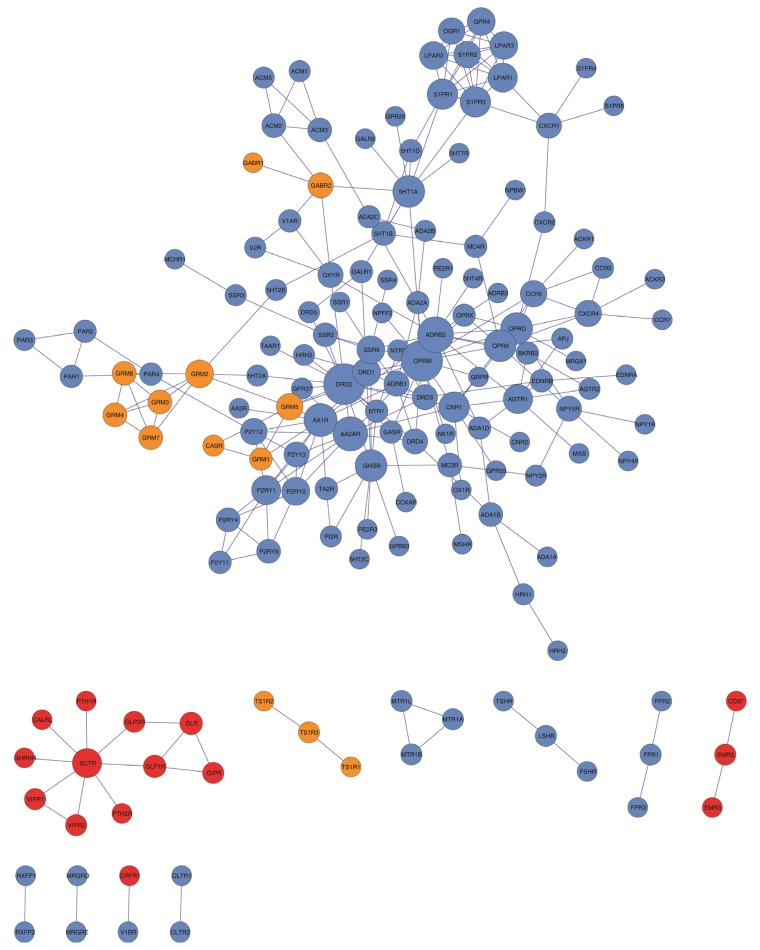
GPCR HetNet graph. Color code: blue, Family 1 or Class A; red, Family 2 or Class B; orange, Family 3 or Class C.

As seen in Table 2 and Figure 1, the GPCR-HetNet is comprised of 156 nodes (protomers) and 260 degrees (interactions). In the network, 44% of the protomers/nodes have three or more interactions. However, nine protomers/nodes have 10 or more connections: OPRM (17), DRD2 (17), ADRB2 (13), AA2AR (12), AA1R (11), OPRK (10), OPRD (10), GHSR (10) and 5HT1A (10). Together these nine highly connected protomers account for 42% of all links in the GPCR HetNet network. In the network the maximal distance between any two protomers (the graph diameter) was nine. A network with a small diameter is often termed a “small world architecture” network in which any two nodes can be connected with relatively short paths. This “small world architecture” effect observed in the GPCR-HetNet, has been detected in several biological systems. It also depends on other network properties like the clustering coefficient or the path length indicator.

**Figure 2.**
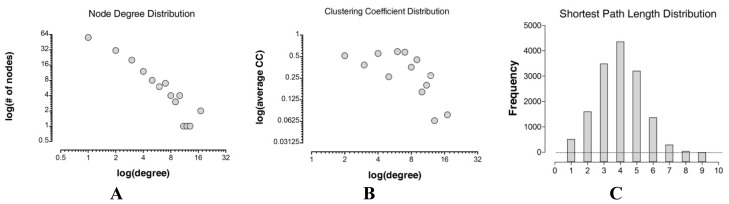
Topological properties distributions. In (**A**) and (**B**) we present the node degree distribution and clustering coefficient distribution for the GPCR receptor–receptor interaction network, respectively. The network shows power-law node degree and clustering coefficient distributions (see Table 3 for further details). Axes are plotted on logarithmic scale. In (**C**) we show the path length distribution of the GPCR-HetNet.

Path length tells us how many edges we need to pass through to travel between two nodes and is a measure of the efficiency of information transfer in the network as well as the overall navigability. The path length distribution for the GPCR-HetNet is shown in Figure 2C. The mean path length is 3.9. Few path lengths fall into the extreme upper categories (path lengths 8 and 9) as compared to the lower extremes (path lengths 1 and 2), indicating that most of the receptors within the network can be linked to every other protomer by a small number of edges. Short paths are considered more desirable because they minimize transition times [66]. One drawback, however, is that they may be highly susceptible to local disturbances which can be transmitted throughout the network quickly.

Based on experimental evidence, we introduced the concept of receptor mosaic (RM) [67]. The idea of RM suggests that receptors can form even more complex and dynamic receptor networks, with respect to time and receptor stoichiometry. Recently, trimeric RMs were described as mGlu_5_–D_2_–A_2A_ receptors [68], as A_2A_–D_2_–CB_1_ receptors [69,70] and as 5-HT1A-GalR1-GalR2 [71]. They have the potential to make a significant contribution to the diversity and specificity of GPCR trafficking and signalling and have been implicated in multiple neurological and psychiatric disorders. In light of new indications from the GPCR-HetNet analysis, which shows that most of the receptors within the network can be linked to every other protomer by a small number of paths, it becomes clear that GPCR heteromers are available in an efficient manner to form higher order heteroreceptor complexes or RM (experimentally demonstrated [68]).

The analysis of the degree of distribution and clustering coefficient approximates a power law that indicates a scale-free topology (Figure 2A,B and Table 3). The scale-free model of the GPCR-HetNet is apparent in Figure 1, where most protomers participate in only a small amount of interactions, but a few participate in dozens. The advantage of this type of organization is that the system is more robust and network properties are often determined by a relatively small number of highly connected protomers/nodes that are known as hubs.

### 2.3. Hubs and Non-Hubs within the GPCR HetNet

The topological analysis reveals that the GPCR-HetNet is not randomly organized but is rather of a “scale-free” format containing hubs with many connections and a large number of nodes that have one or a small number of connections.

We also now know, based on various proposed models aimed to explain the development of the scale-free topology of the protein–protein interaction network during evolution, that in such a particular architecture the probability that a newly added node interacts with an existing node is proportional to its connection degree. This leads to a so-called preferential attachment model in which rich nodes get richer during evolution and finally form a scale-free network [6,7]. In general, core components of a network tend to be conserved, whereas components at the periphery or false interactions are not. Therefore, hub components in a scale-free network are extremely important and hence usually play essential roles in biological systems *versus* lesser-connected nodes [6,72–74]. We can also state that the current identified hubs, because they are part of a “scale-free” network, can continuously exist as hubs independently of the evolution or growth of the network when new nodes (protomers) are identified and added in the feature.

**Figure 3.**
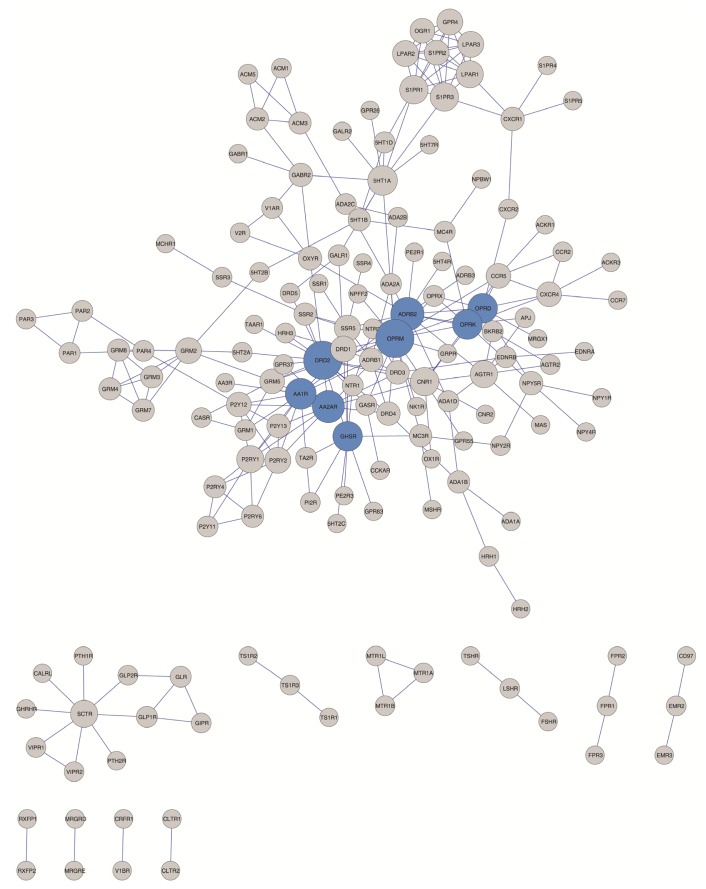
Hubs identified according to different criteria (“top 95% of the high degree nodes” and “node degree > 8”) are shown in blue.

The hub designation itself, however, is somewhat arbitrary. For clarity, hubs are defined in this work following four different criteria including the more objective characterization of hubs described recently by Vallabhajosyula *et al.*, 2009 [75] (see the Experimental Section and Table 5). As shown in Tables 4 and 5 and Figure 3, two of the hub definitions employed (the top 95% of the high degree node criterion [76] and a node degree higher than eight interaction criterion [77]) allow the identification of eight hubs (OPRM, DRD2, ADRB2, AA2AR, AA1R, OPRK, OPRD and GHSR) which belong to the rhodopsin-like class A GPCR subfamily. A more permissive criterion, where the node degree is higher than five interactions [72] results in the identification of 29 hubs, among which at least two hubs belong to the Class C GPCR subfamily (mGluR2 and mGluR5) and one to the Class B GPCR subfamily (SCTR). However, the relative connectivity criterion [75] in the GPCR-HetNet identified as a hub only the dopamine D2 receptor (DRD2), the beta-2 adrenergic receptor (ADRB2) and the mu-type opioid receptor (OPRM). When considering the less conservative criterion some, if not all of these protomers, may eventually emerge as hubs in the evolution of the GPCR-HetNet when the interaction profiles expand.

Previous work also indicated that the D2 receptor is a hub receptor in view of the existence of a large number of different types of D2 heteroreceptor complexes in the Central Nervous System (CNS) [14,78]. The Tarakanov and Fuxe hypothesis [79] states that protriplet homologies participate in recognizing the other receptor protomer of the heteromer via postulated “guide-and-clasp” interactions in the receptor interface [79–82]. As to the D2 receptor, the AVI protriplet homology may participate in receptor–receptor interactions of five D2 receptor heteromers: D1–D2, D2–GPR37, D2–5HT2A, D2–CCK2R and D2–NMDA. The DLL protriplet homology is present in six D2 receptor heteromers, D1–D2, D2–D3, D2–D4, D2–CCK2R, CB1–D2 and D2–NTS1, but located in part in different types of D2 receptor heteromers compared with the AVI triplet [78]. Thus, the D2 receptor emerges as a hub receptor in the receptor networks of the CNS, which via allosteric receptor–receptor interactions in large numbers of D2 heteroreceptor complexes play a major integrative role. The impact of the D2 receptor on information handling is also demonstrated by the fact that it is the major target for antipsychotic drugs [83].

### 2.4. Clustering and Modularity within the GPCR HetNet

**Figure 4.**
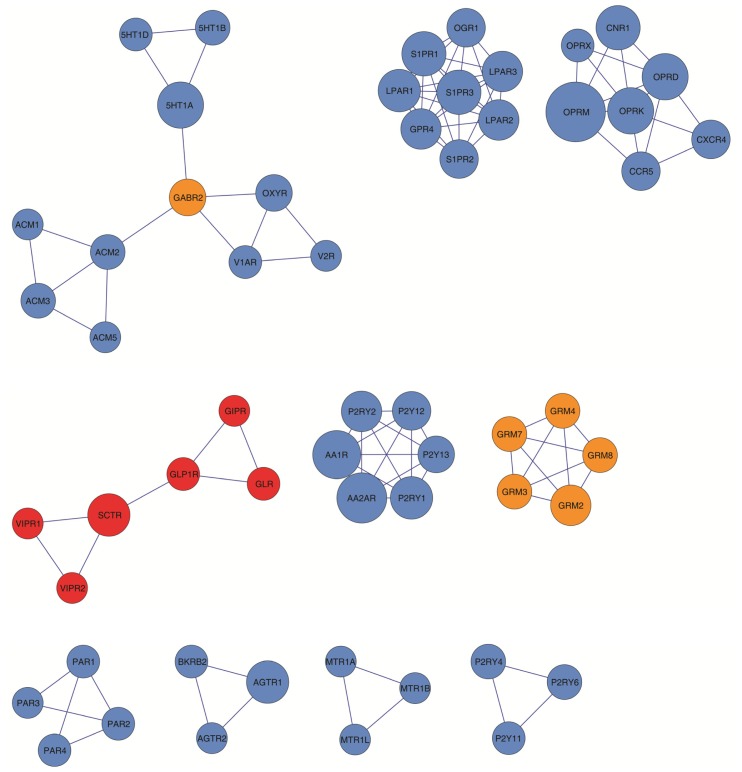
GPCR-HetNet motifs. Nine clusters identified using the MCODE search algorithms are represented and rank ordered, from top left to right down, according to their density (inter-connectivity) and size (number of protomers).

Visual inspection of the GPCR-HetNet (Figure 1) shows that rhodopsin-like receptors (blue), especially monoaminergic receptors, most generally occur in the dense regions of the graph. However, the rhodopsin-like members, sphingosine 1-phosphate receptors (S1P receptors) and lysophosphatidic acid receptors (LPA receptors) (top-right of the graph), are interacting with several GPCRs but are connected to the network by a relatively small number of links. Members of the metabotropic glutamate receptor family (orange) border the central area but are situated in less dense regions. The Secretin-like receptor family (red), despite being well connected and represented in this network with almost 60% of their members, does not have connectivity to the rest of the network. There are also several GPCRs (23 protomers) that are not connected to the main network at all. These are: TSHR, LSHR, FSHR, MTR1A, MTR1B, MTR1L, FPR2, FPR1, FPR3, CD97, EMR2, EMR3, TS1R2, TS1R3, TS1R1, CLTR1, CLTR2, CRFR1, V1BR, MRGRE, MRGRD, RXFP1, RXFP2. The lack of connectivity for these proteins may be due to the absence of experimental data. However, they may represent future branch points or subgraphs like, for example, the SCTR or MTR1A clusters (see Figure 4). As mentioned above, some receptors are less widely expressed in some tissues or organs than others, or have a more restricted tissue expression profile, and as a result allow their members to have a lower probability of encounters and interactions and therefore lower heterodimeric diversity.

Receptor function is likely to be carried out in a highly modular manner, and GPCRs are not the exception. From the point of view of the GPCR-HetNet, modularity refers to a group of physically or functionally linked protomers (nodes) that work together to achieve a distinct function. In a network representation, a module (cluster) appears as a highly interconnected group of nodes that can be determined by the clustering coefficient, the signature of a network’s potential modularity. The clustering coefficient quantifies the number of connected pairs between a node and its neighbours and can be measured both globally (the average of the clustering coefficients for all the nodes in the entire network) and locally (the embeddedness of single nodes). It is important because it can provide insight into the overall organization of the relationships within a network (network hierarchical character). It may also indicate the presence of physical/functional modules which, in the case of receptor–receptor interaction network, can represent higher order heteroreceptor complexes or receptor mosaics. The global clustering coefficient of the GPCR-HetNet (the average of the clustering coefficients for all nodes in the network) is 0.25. Using a cluster search algorithm that considers highly interconnected dense regions within a network (MCODE) 10 clusters were identified in the GPCR-HetNet (Figure 4). Out of 156 total protomers in the network, 56 (36%) of these are located within clusters.

## 3. Experimental Section

### 3.1. GPCR Receptor–Receptor Interaction Dataset

Interaction data for each of the individual human GPCR protomers were obtained manually from the Search Tool for the Retrieval of Interacting Genes [84] database and literature (SCOPUS database). Only protomers that have been validated by one (65%) or more (35%) independent publication (experimentally verified interactions) were used to create the graph. If we, in addition to the number of publication criteria, also consider the number of experimental methods used to validate each pair, the analysis of the 260 pairs revealed that more than 96% of them have been validated by more than one of the following experimental methods (co-immunoprecipitation, BRET/FRET/SRET/TR-FRET/BiFC, *in situ* PLA, and FCCS). Also, from the total of 260 pairs, only 15 pairs (5.78%) represent a controversial issue. Looking carefully at the publications on this controversial approximately 6%, 3% do not represent a true controversy. For instance, the P2RY2/P2RY4 and P2RY2/P2RY6 are heteromer pairs that can interact in different cell types but do not interact in PC12 cells. The controversy does not rely on the interaction itself but on the type of cell in which it can take place. It is reasonable to think that depending on the tissue or cell type, many GPCR heteromers will show a different pattern of receptor–receptor interactions, dependent on the cell milieu. The second false controversy is the example of D2R-D4R, where the controversy arises from the receptor isoform studied. D2LR interacts with the main D4R isoforms but D2SR only interacts with some of them. Variability in the intracellular loops of GPCR, as a result of gene splicing, may play an important role in the selectivity and affinity of GPCR protomers.

The dataset list and the references for each interaction are provided at GPCR-HetNet [60], containing 260 pairs of GPCR heteromers taken from a total of 156 unique GPCR protomers which represent approximately 20% of the total number of human GPCR protomers. This database will be updated as needed and relies on the continued support of the GPCR community.

### 3.2. Network Construction and Analysis

Networks have proven to be a useful mathematical artifact for studying complex systems in multiple disciplines such as economics, sociology, and biology [2,6,8,9]. The term biological network refers to networks that describe relationships among a set of elements within biological systems. A network can be formally defined by a set of *N* elements and a set of *R* relations among those elements. A graph is a common way of visualizing networks. In a network graph, the nodes of the graph are the elements of the network and the edges correspond to the relations among the elements of the network. The GPCR-HetNet can be considered as a network of interactions amongst GPCR protomers. Each protomer in a receptor complex is considered as a node in the network and the connections between the nodes are the edges. The interaction data were used to build and analyze the network using Cytoscape [85], a network visualization and analysis platform that supports a wide variety of plug-ins relative to network analysis and manipulation. Duplicated edges and self-loops resulting from reciprocal interaction detection (ex. homodimerization) were removed prior to the analysis. The network was treated as undirected throughout the study, meaning that there were no distinctions implied between the nodes. An undirected network or graph is a network where all the edges (the connection between nodes) are bidirectional. In contrast, a network where the edges point in a direction is called a directed network. It is worth mentioning that one network may be depicted by different graphs. This means that the same network can have different layouts or shapes, which, in turn, may lead to inconsistent interpretations depending on the chosen graph. In order to overcome this misinterpretation issue, many studies propose determining topological features of networks instead of analyzing their graphical representations [6,7,86]. A topological feature of a network is an invariant property whose value is the same no matter the network graph chosen. Cytoscape was used to calculate the basic network metrics such as the number of nodes and edges, density, diameter, degree distribution, path length, and clustering coefficient. Hubs were identified using four different criteria (see below for more details).

### 3.3. Topological Features

The most elementary topological feature of a network is the node *degree_k_*, which measures the number of connections or links the node maintains with the other nodes. The node *degree distribution* of a network, *P*(*k*), is the fraction of nodes that have exactly *k* connections to other nodes, *i.e.*, *P*(*k*) *= degree_k_*/*N*, where *degree_k_* is the number of nodes with degree equal to *k*. A *path* between a pair of nodes is a set of adjacent edges and nodes that we need to visit in order to travel from one node to the other. The *path distance* is the number of edges the path contains. The *shortest path* between a pair of nodes is the path that has the smallest path distance. *A clustering coefficient* assesses the trend of the nodes of the network to form clusters. The local clustering coefficient of node *n* is given by the following formulae: *C*(*n*) *= 2n_l_*/*k* (*k* − 1), where *n_l_* is the number of connections among the nodes that *n* is connected to. The clustering coefficient of a network is the average of the local clustering coefficients of all nodes in the network. The *clustering coefficient distribution*, *C*(*k*), is defined by the average of the clustering coefficient of the nodes with degree equal to *k*. The so-called *network density D* assesses how connected the network is, *D = average_k_*/*N* − 1, where *average_k_* is the average degree of the network. A *connected component* is a subgraph in which every pair of nodes is connected to each other by at least one path. Another feature of the topological connectivity of a network measures the relative size of the largest component of a network. This is computed by dividing the number of nodes in the largest component by the number of total nodes in the network. This measure is referred to as the *relative connectivity*, *f* (for further details see [6]).

### 3.4. Network Models

Knowing the model of our biological network is essential to further understand the complex system that is modeled. To this aim we need to examine two of the aforementioned topological features: node degree distribution, *P*(*k*), and clustering coefficient, *C*(*k*). Barabási *et al.* describe three models of biological networks labelled as: random, scale-free, and hierarchical [6]. In a random network, *P*(*k*) follows a Poisson distribution and *C*(*k*) is independent of the node degree *k*. In other words, the majority of nodes have roughly the same number of connections, and their tendency to form clusters is the same no matter the node degree. In contrast, node degrees show a power-law distribution, *P*(*k*)*~k*^−γ^*,* in scale-free and hierarchical models. In these models, networks have many nodes with small degrees and allow nodes with high degrees. The most notable characteristic of these two models is the so-called preferential attachment property, which implies that a newly added node is more likely to interact with nodes of higher degrees. Scale-free and hierarchical models, however, differ from each other in the way *C*(*k*) is expressed. Similar to random networks, *C*(*k*) is independent of *k* in scale-free networks. In contrast to scale-free networks, *C*(*k*) in hierarchical networks can be expressed as a function of the degree in the following way: *k*^−1^. Hierarchical networks can be seen as a special type of scale-free network with a large clustering coefficient.

### 3.5. Hubs and Non-Hubs

Preferential attachment property in scale-free and hierarchical models leads to the origin of hubs, a relatively small set of highly connected nodes. Several studies report that hubs have biological significance in biological networks such as protein interaction networks [72–74]. However, despite its simple definition, there is no consensus on when a node is a hub. In Batada *et al.* the top 95% of the high degree nodes were selected as hubs [76]. Nodes with degrees greater than five and eight were defined as hubs in Han *et al.* [72] and Ekman *et al.* [77], respectively. Vallabhajosyula *et al.* [75] proposes an objective characterization of hubs, which relies on the idea that hubs have lower connectivity among themselves than non-hub nodes. The procedure for selecting hubs according to relative connectivity is as follows. First, we create a systematic list of the network nodes by the decreasing order of their degree. Secondly, we generate successive subgraphs adding, each time, one node from the degree list. For instance, we first generate *G*_1_, which consists of just one node: the one that appears at the front of the degree list. Then we add the second node from the list and generate subgraph *G*_2_, and so on. For each subgraph we compute its relative connectivity *f*. This process continues until we obtain a subgraph *G_k_* whose relative connectivity *f_k_* is larger than *f_k_*_−1_. Value *k* is interpreted as the natural boundary between hub and non-hub nodes, and nodes from *G_k_*_−1_ are the hubs of the network. In this paper we identify hubs following all these four selection criteria.

### 3.6. Clusters and Motif

Clusters were found with Molecular Complex Detection (MCODE) [87] using the haircut option which identifies nodes that have limited connectivity at the cluster periphery. A value of 2.0 was used for the degree of cutoff, representing the minimum number of edges for a node to be scored. The *K*-Core value, which is used to filter out clusters lacking a maximally interconnected core, was specified for two edges [66].

## 4. Conclusions

The existence of GPCR heteromers was demonstrated through diverse methods [12,17,52–54]. In this study, we manually collected static/non-dynamical human GPCR data derived from these interaction studies in annotated STRING and SCOPUS databases. We further integrated the relationship information in a large-scale graph, called the GPCR heteromer network [60], where the vertices are the receptor protomers and the edges are their relationships. The results for the GPCR-HetNet indicate a scale-free model in which a few of the protomers dominate the connectivity and hold the network together. Three different hub criteria show that the dopamine D2 receptor (DRD2), the beta-2 adrenergic receptor (ADRB2), the growth hormone secretagogue receptor type 1 (GHSR), the mu-type opioid receptor (OPRM), the delta-type opioid receptor (OPRD), the kappa-type opioid receptor (OPRK), the adenosine A2A receptor (AA2AR) and the adenosine A1 receptor (AA1R) are the hubs in the network. Other highly connected protomers are also identified and described in this study, as well as the emergence of potential allosteric mechanism avenues and higher order heteroreceptor complexes. In this study we present for the first time the overall architecture of the GPCR heteromers. The GPCR-HetNet provides insight into receptor–receptor interaction connectivity, topology, and organization that could be used to generate plausible hypotheses and help researchers to better understand GPCR heteromer systems and design experiments.

## Figures and Tables

**Table 1.** General properties of the receptor–receptor heteromer interaction network. F1, G-protein coupled receptor 1 (Class A or rhodopsin-like); F2, G protein-coupled receptor 2 (Class B or Secretin receptor family); F3, G protein-coupled receptor 3 (Class C or Metabotropic glutamate/pheromone).

Number of receptor protomers per GPCR family that showed to form at least one heteromer			Number of receptor interaction pairs/connectivity (intrafamily: F1, F2, F3 and interfamily)			
F1	F2	F3	F1	F2	F3	Interfamily
128	15	13	219	15	17	9

**Table 2.** GPCR HetNet topological metrics.

Protomers/nodes	Interactions/edges	Density	Diameter	Average degree	Clustering coefficient
156	260	0.02	9	3.03	0.25

**Table 3.** Network model comparison. *R*^2^ as metric of goodness of distribution fit.

Node degree distribution		Clustering coefficient distribution	
Linear	Power-law	Linear	Power-law
0.53	0.91	0.56	0.80

**Table 4.** No-hubs and hub selection criteria.

No. hubs (degree = 1)	Hub selection criteria							

	Relative connectivity		Top 95% of the high degree nodes		Node degree > 5		Node degree > 8	
	cutoff	#hubs	cutoff	#hubs	cutoff	#hubs	cutoff	#hubs
57	12	3	10	8	5	29	8	12

**Table 5.** Chosen non-hubs and hubs.

Non-hubs			
5HT2B, 5HT2C, 5HT4R, AA3R, ACKR1, ACKR3, ADA1A, ADRB3, CALRL, CCKAR, CCR7, CD97, CLTR1, CLTR2, CNR2, CRFR1, EDNRA, EMR3, FPR2, FPR3, FSHR, GABR1, GALR2, GHRHR, GPR26, GPR55, GPR83, GRPR, HRH2, MAS, MCH1R, MRGRD, MRGRE, MRGX1, MSHR, NK1R, NPBW1, NPFF2, NPY1R, NPY4R, OX1R, PE2R1, PE2R3, PTH1R, PTH2R, RXFP1, RXFP2, SIPR4, S1PR5, SSR1, SSR4, TAAR1, TS1R1, TS1R2, TSHR, V1BR			

**Hub selection criteria**			

**Relative connectivity**	**Top 95% of the high degree nodes**	**Node degree > 5**	**Node degree > 8**

DRD2 = 17,	DRD2 = 17,	DRD2 = 17,	DRD2 = 17,
OPRM = 17,	OPRM = 17,	OPRM = 17,	OPRM = 17,
ADRB2 = 13	ADRB2 = 13,	ADRB2 = 13,	ADRB2 = 13,
	AA2AR = 12,	AA2AR = 12,	AA2AR = 12,
	AA1R = 11,	AA1R = 11,	AA1R = 11,
	OPRK = 10,	OPRK = 10,	OPRK = 10,
	OPRD = 10,	OPRD = 10,	OPRD = 10,
	GHSR = 10	GHSR = 10,	GHSR = 10,
		5HT1A = 10,	5HT1A = 10,
		S1PR3 = 9,	S1PR3 = 9,
		S1PR1 = 9,	S1PR1 = 9,
		CNR1 = 9,	CNR1 = 9
		SCTR = 8,	
		P2RY1 = 8,	
		LPAR1 = 8,	
		AGTR1 = 8,	
		SSR5 = 7,	
		P2RY2 = 7,	
		LPAR3 = 7,	
		LPAR2 =7,	
		GRM2 = 7,	
		GPR4 = 7,	
		DRD1 = 7,	
		S1PR2 = 6,	
		P2Y12 = 6,	
		OGR1 = 6,	
		GRM5 = 6,	
		CXCR4 = 6,	
		CCR5 = 6	
